# Supplementary material for: All-at-once RNA folding with 3D motif prediction framed by evolutionary information
Source: Nat Methods. 2025 Oct 3;22(10):2094–106. doi: 10.1038/s41592-025-02833-w (PMC12510878; doi:10.1038/s41592-025-02833-w)
Supplement: Supplementary file 3 — Source code to run CaCoFold-R3D. Data and results. [file 41592_2025_2833_MOESM3_ESM.gz › supplemental_material/results/CaCoFold-R3D/CaCoFold-R3D_Rfam_vshuffle/RF01879_TUSC7.R2R.sto.pdf]

RF01879_TUSC7

5′-A C A A G C Y A A G A G A U A C A A A G G G A A A A G U G U G A C U G C U C U G A C A G C A A A A A C A A G C A A A U A U C U C C A G A G G G G A U U A R U C C Y U C U G C U U C A A A G U A A C Y A A A A C A A A G A R A G C U G A G C C A G C U U C ● C U G G R A A C A R C A C C U G U C C A U C A G A C A A G G A U G A G C U A A U
